# New 18(4→3)*-Abeo*-Abietanoids from *Tripterygium wilfordii*

**DOI:** 10.3390/molecules23102467

**Published:** 2018-09-26

**Authors:** Lin Ni, Ying-da Zang, Jing-zhi Yang, Chuang-jun Li, Jie Ma, Dong-ming Zhang

**Affiliations:** 1 State Key Laboratory of Bioactive Substance and Function of Natural Medicines, Institute of Materia Medica, Chinese Academy of Medical Sciences and Peking Union Medical College, Beijing 100050, China; nilin_fjau@126.com (L.N.); zydaitfj@imm.ac.cn (Y.-d.Z.); yjzh@imm.ac.cn (J.-z.Y.); lichuangjun@imm.ac.cn (C.-j.L.); majie@imm.ac.cn (J.M.); 2 College of Plant Protection, Fujian Agriculture and Forestry University, Fuzhou 350002, China

**Keywords:** *Tripterygium wilfordii*, tripordolide, X-ray, NO

## Abstract

Three 18(4→3)*-abeo*-abietanoids, a new natural product and two new compounds, named tripordolides A–C (**1**–**3**), were isolated from the leaves of *Tripterygium wilfordii.* Their structures were elucidated on the basis of their spectroscopic analysis, and the absolute configuration of compounds was confirmed by CD and X-ray crystallographic analysis using anomalous scattering of Cu *K*α radiation. Compounds **1** and **3** showed moderate inhibitory activities against NO production in lipopolysaccharide-induced (LPS) RAW 264.7 macrophages in vitro.

## 1. Introduction

**Figure 1:**
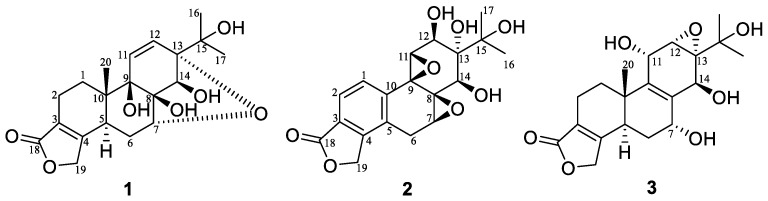
The structures of compounds **1**–**3**.

*Tripterygium wilfordii,* also called “leigongteng”, is a member of the Celastracrae family [1], and was commonly used in Traditional Chinese Medicines to cure IgA nephropathy cancer, systemic lupus erythematosus, ankylosing spondylitis, psoriasis, and idiopathic diseases [2,3,4]. Earlier chemical studies on *T. wilfordii* have led to the isolation of alkaloids [5,6,7], diterpenes [8], triterpenes [9], sesquiterpenes [10,11,12], and lignans [13], which showed a wide range of biological activity, including anti-fertility, anti-inflammatory, immunosuppressive, anti-HIV, and anti-tumor [14]. In order to explore structurally and biologically interesting natural products from *T. wilfordii*, three new 18(4→3)*-abeo*-abietanoids (Figure 1), containing a new natural product and two new compounds, named tripordolides A–C (**1**–**3**), were isolated. Herein, we describe the isolation, structural elucidation, and activities of these compounds.

## 2. Results and Discussion

**Figure 2:**
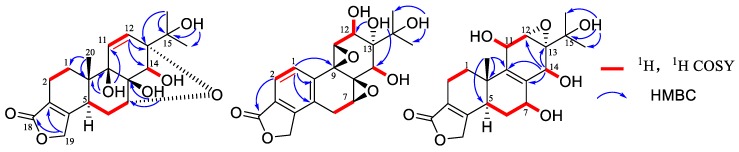
Key ^1^H,^1^H COSY and HMBC correlations of compounds **1**–**3**.

Tripordolide A (**1**) was obtained as a colorless needle, and gave a positive reaction in the kedde reagent test on the TLC, which suggested the structure of compound **1** contained the lactone ring. Its molecular formula of C_20_H_26_O_7_ was determined on the basis of HRESIMS at *m/z* 379.17532 [M + H]^+^ (calculated for C_20_H_27_O_7_: 379.17513), indicating eight degrees of unsaturation. The ^1^H NMR spectrum data of **1** (Table 1) exhibited signals of three methyl groups [*δ*_H_ 1.12 (6H, s, H_3_-16 and H_3_-17)], two aromatic methines [*δ*_H_ 6.10 (1H, d, overlap) and 6.12 (1H, d, overlap)], an oxygenated methylene [*δ*_H_ 4.77(2H, m)], and two oxygenated methines [*δ*_H_ 3.96(1H, d, *J* = 2.8 Hz, H-7; 3.99 (1H, d, *J* = 6.0 Hz)]. The ^13^C NMR and DEPT spectra demonstrated 20 carbons resonances, which contained a carbonyl, seven oxygenated carbons (four quaternary carbons, a methylene, and two methines), two aromatic quaternary carbons, and two aromatic methenes. According to this information, the skeleton of **1** was recognized to be an 18(4→3)*-abeo*-abietane [15], which contained five rings and four hydroxy groups to be satisfied with its indices of hydrogen deficiency. Its planar structure and ^1^H, ^13^C NMR assignments could be confirmed with an analysis of the two-dimensional NMR (Figure 2). A ring of α, β-unsaturated-γ-lactone was confirmed based on the HMBC correlation from H_2_-19 [*δ*_H_ 4.77 (2H, m)] to C-18(*δ*_C_ 173.5) and C-3(*δ*_C_ 122.8). Structure of rings A and B could be determined by the ^1^H, ^1^H COSY correlations of H-1/H-2 and H-5/H-6/H-7, and the HMBC correlation of H_3_-20 [*δ*_H_ 1.00 (3H, s)]/C-1 (*δ*_C_ 27.5), C-5 (*δ*_C_ 35.1), C-9 (*δ*_C_ 76.0), and C-10 (*δ*_C_ 40.2). The HMBC Correlations from H-12 to C-9, C-13 (*δ*_C_ 82.7), C-14 (*δ*_C_ 77.6), and C-15 (*δ*_C_ 70.5); from H-14 [*δ*_H_ 3.99 (1H, d, *J* = 6.0 Hz)] to C-8 (*δ*_C_ 75.9) and C-13 corroborated the ring C. The four hydroxy groups were assigned to C-8, C-9, C-14, and C-15, indicated by the HMBC correlations of 8-OH [*δ*_H_ 4.86 (1H, s)]/C-7 (*δ*_C_ 74.3) and C-8, 9-OH [*δ*_H_ 4.03 (1H, s)]/C-10, C-11 (*δ*_C_ 134.0), and C-12 (*δ*_C_ 131.1), 15-OH [*δ*_H_ 4.15 (1H, s)]/C-13, C-15, C-16 (*δ*_C_ 24.3), and C-17 (*δ*_C_ 27.4); the ^1^H, ^1^H COSY correlation from H-14 to 14-OH [*δ*_H_ 4.90 (1H, d, *J* = 6.0 Hz)]. Finally, the fifth ring was an oxide bridge between C-7 and C-13 (furan ring) to meet with the HRESIMS data.

**Figure 3:**
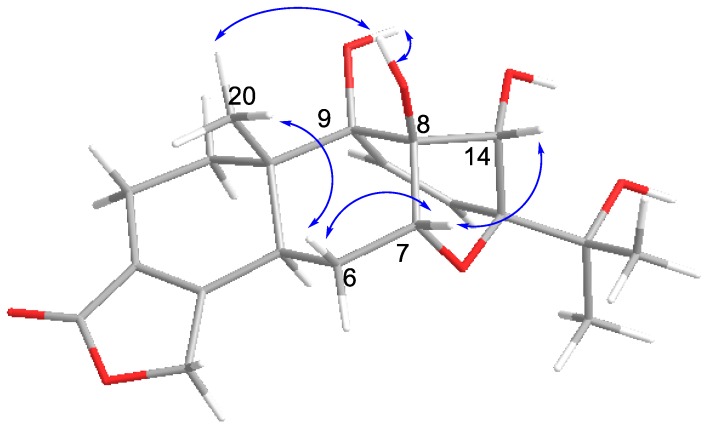
Key NOESY correlations of compound **1**.

**Figure 4:**
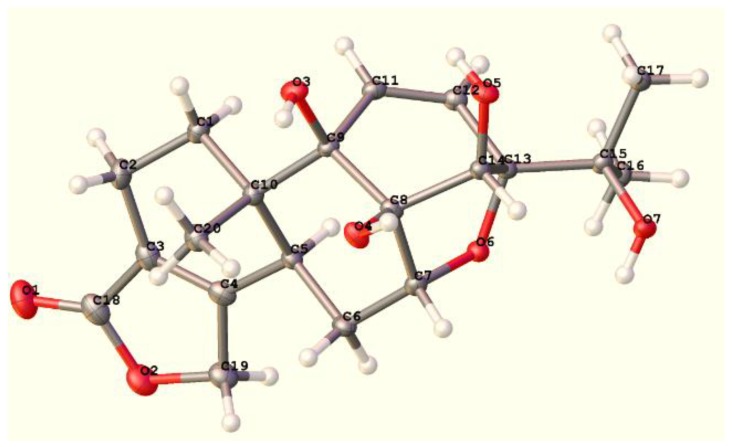
Single crystal structure of compound **1**.

NOESY analysis was used to deduce the relative configuration of **1** (Figure 3). Orientations were confirmed by the NOE correlations of H_3_-20/8-OH, 9-OH and H-6β; of H-6β /H-7/H-14. Finally, the proposed structure of **1** was confirmed by an X-ray crystallography analysis using anomalous scattering of Cu *K*α radiation (Figure 4). Accordingly, the absolute configuration of 5*S*, 7*R*, 8*S*, 9*R*, 10*S*, 13*S*, and 14*S* was established based on the value of the Flack absolute structure parameter—0.02(11).

**Figure 5:**
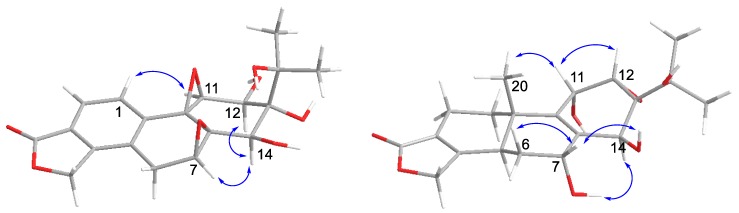
Key NOESY correlations of compounds **2** and **3**.

Tripordolide B (**2**) was obtained as a colorless oil and showed a [M + Na]^+^ ion peak at *m/z* 399.1052 (calculated for 399.1050) in the HRESIMS, which corresponded to the molecular formula C_19_H_20_O_8_. Some obvious signals, a ring of α, β-unsaturated-γ-lactone [*δ*_H_ 5.42 (2H, m, H-17); *δ*_C_ 170.4 (C-18), 68.9 (C-19), 124.4 (C-3), and 146.5 (C-4)] and an oxygen isopropyl group [*δ*_H_ 1.23, 1.41 (each 3H, s, H_3_-16 and H_3_-17); *δ*_C_ 25.2(C-16), 26.8 (C-17), and 73.6 (C-15)], indicated **2** was also an 18(4→3)-abeo-abietane derivative (Table 1) and similar to a less 20-CH_3_ and aromatized nor-abietane, tripterlide F [16]., This was proven by the HMBC correlations of H-1 [*δ*_H_ 7.22 (1H, d, *J* = 8.0 Hz)]/C-4, C-5 (*δ*_C_ 128.7) and C-9 (*δ*_C_ 60.7); of H-2 [*δ*_H_ 7.70 (1H, d, *J* = 8.0 Hz)]/C-4, C-10 (*δ*_C_ 138.5) and C-18 (Figure 2). Some major differences between the two compounds in the chemical shift values of C-12 and C-13 were observed, which could be attributed to the breakage of a trisubstituted epoxide between C-12 (*δ*_C_ 69.7) and C-13 (*δ*_C_ 74.9). Furthermore, compared to the tripterlide F, the oxygen isopropyl group of **2** also lead to the lower field of C-15 (*δ*_C_ 73.6), C-16 (*δ*_C_ 25.2), and C-17 (*δ*_C_ 26.8). The relative configuration was established by the NOESY correlations of H-12/H-1 and of H-7/H-14/H-12 (Figure 5). Thus, **2** was elucidated as shown, named tripordolide B.

Tripordolide C (**3**) was obtained as a colorless oil and had the molecular formula C_20_H_26_O_7_, deduced from the [M + Na]^+^ ion peak at *m/z* 401.1574 (calculated 401.1571). Its ^1^H and ^13^C NMR characteristics (Table 1) could be concluded; **3** was also an 18(4→3)-abeo-abietane derivative. Its planar structure has been confirmed by an analysis of a two-dimensional NMR (Figure 2). HMBC correlation of 15-OH [*δ*_H_ 4.17 (1H, s)]/C-15 (*δ*_C_ 69.6), C-16 (*δ*_C_ 27.4), and C-17 (*δ*_C_ 26.1); ^1^H, ^1^H COSY correlations of 7-OH [*δ*_H_ 5.34 (1H, d, *J* = 5.4 Hz)]/H-7 [*δ*_H_ 4.22 (1H, m)], 11-OH [*δ*_H_ 5.02 (1H, d, *J* = 8.0 Hz)]/H-11[*δ*_H_ 4.45 (1H, br d, *J* = 8.0 Hz)], and 14-OH [*δ*_H_ 4.86 (1H, d, *J* = 4.4 Hz)]/H-14 [*δ*_H_ 4.47 (1H, d, *J* = 4.4 Hz)] suggested that the four hydroxy groups were located at C-7, C-11, C-14, and C-15. HMBC correlations from H_3_-20 [*δ*_H_ 1.05 (1H, s)] to C-5 (*δ*_C_ 40.8), C-10 (*δ*_C_ 35.6), and C-11 (*δ*_C_ 62.0), and from H-14 [*δ*_H_ 4.47 (1H, d, *J* = 4.4 Hz)] to C-8 (*δ*_C_ 131.7), C-9 (*δ*_C_ 137.8), C-12 (*δ*_C_ 55.9), and C-13 (*δ*_C_ 61.9) indicated a double bond between C-8 and C-9. Moreover, a trisubstituted epoxide between C-12 and C-13 was formed to meet with its indices of hydrogen deficiency, which also led the chemical shift of C-12 (*δ*_C_ 55.9) and C-13 (*δ*_C_ 61.9) to upfield. The relative configuration was established by the NOESY correlations of H-12/H-11/H-20 and of H-6β/H-7/H-14-OH (Figure 5). Therefore, **3** was elucidated as shown, named tripordolide C.

Three 18(4→3)*-abeo*-abietanes were evaluated for their inhibitory activities against NO production in lipopolysaccharide-induced (LPS) RAW 264.7 macrophages in vitro. Compounds **1** and **3** inhibited NO production in mouse peritoneal macrophage 48.4 ± 5.7% and 53.5 ± 4.8% at a concentration of 10 μM, while the positive control dexamethasone gave an inhibitory ratio of 63.1 ± 4.6% at an identical concentration.

## 3. Experimental Section

### 3.1. General

A Bruker APEX DUO diffractometer with Cu Kα radiation ((Bruker-Biospin, Billerica, MA, USA) was used to collect the X-ray data of compound **1**. A JASCO P-2000 polarimeter (JASCO, Easton, MD, USA), JASCO V650 (Thermo Scientific, Waltham, MA, USA), spectrophotometer, and XT-5B micro melting point apparatus (Aihuishi Ltd. Shanghai, China) were used to collect optical rotations, UV spectra, and melting points, respectively. VNS-600 and Bruker-500 (Bruker-Biospin, Billerica, MA, USA) spectrometers were used to collected NMR spectra. The Agilent 1100 series LC/MSD ion trap mass spectrometer (Agilent, Waldbronn, Germany) was used to collect HRESIMS spectra. Preparative HPLC was performed on a Shimadzu LC-6AD instrument with a SPD-20A detector (Shimadzu Corp., Tokyo, Japan), using a YMC-Pack ODS-A column (2 × 25 cm, 5 μm, YMC, Tokyo, Japan). Column chromatography was performed with silica gel (200−300 mesh, Qingdao Marine Chemical Inc., Qingdao, China), polyamide (60–100 mesh, Changfeng Chemical Inc., Jiangsu, China), and ODS (50 μm, YMC, Tokyo, Japan). 

### 3.2. Plant Material

The leaves of *T. wilfordii* were collected in Taining, Fujian, China, in September 2009 and identified by Professor Lin Ma from the Institute of Materia Medica, Chinese Academy of Medical Sciences and Peking Union Medical College. A voucher specimen (No. 20090034) is deposited at the herbarium of the Institute of Materia Medica, Chinese Academy of Medical Sciences, and Peking Union Medical College, China.

### 3.3. Extraction and Isolation

The air-dried and powdered leaves of *Tripterygium wilfordii* (100 kg) were extracted two times with 80% EtOH (800 L) at 80 °C for 2 h. After filtration and evaporation ethanol under reduced pressure at 50 °C, the aqueous residue was diluted with H_2_O and then partitioned three times with ethyl acetate (30 L). The remaining water extract was subjected to passage over polyamide by elution with water and 30%, 60%, and 95% EtOH-H_2_O. Then the water elution was subjected to passage over D101 macroporous resin by elution successively with H_2_O, 30%, 60%, and 95% EtOH-H_2_O (Fractions B_1_-B_4_). Fraction B_3_ (338.5 g) was subjected to passage over a bergmeal column eluting successively with ethyl acetate, ethanol, and methanol (Fractions C_1_–C_3_). Fraction C_2_ (151.3 g) was chromatographed on a silica gel column eluting successively with a solvent gradient system (CHCl_3_−MeOH, 15:1−0:1) to afford 7 fractions (D_1_−D_7_). Fraction D_4_ (3.36 g) was passed over an PRP-512A macroporous resin column with MeOH-H_2_O (10%, 30%, 60% and 100%) to give four fractions (E_1_–E_4_), and the fraction E_3_ (0.45 g) was purified by preparative HPLC (MeOH-H_2_O, 35:65, *v/v*, detected at 210 nm, 8 mL/min) to give **1** (5.3mg), **2** (1.3 mg), and **3** (1.0 mg).

### 3.4. Spectral Data

Single crystals of C_20_H_26_O_7_ were recrystallized from MeOH mounted in inert oil and transferred to the cold gas stream of the diffractometer.

Crystal data of tripordolide A (**1**): C_20_H_26_O_7_, M = 378.41, monoclinic, *a* = 7.7259(3) Å, *b* = 7.3200(3) Å, *c* = 15.4365(5) Å, β = 98.081(3)°, *U* = 864.33(5) Å3, *T* = 105(2), space group P21 (no. 4), Z = 2, μ(Cu Kα) = 0.912, 6799 reflections measured, 3061 unique (*R_int_* = 0.0282) which were used in all calculations. The final *wR*(*F*_2_) was 0.0901 (all data). Flack parameter was −0.02(11).

Crystallographic data for the structure of tripordolide A (**1**) have been deposited in the Cambridge Crystallographic Data Centre (deposition numbers: CCDC 1844652). Copies of the data can be obtained free of charge via www.ccdc.cam.ac.uk.

#### Characterization Data of Tripordolides A–C (**1**–**3**)

*Tripordolide A* (**1**): colorless needle; [α]${}_{D}^{25}$—32.5 (*c* 0.1 MeOH); UV (MeOH) *λ*_max_ (log *ε*) 220 (3.53) nm; ^1^H NMR (DMSO-*d*_6_, 500 MHz) and ^13^C NMR (DMSO-*d*_6_, 125 MHz), see Table 1; HRESIMS *m/z* 379.17532 [M + H]^+^ (calculated for C_20_H_2__7_O_7_, 379.17513). (Supplementary Materials).

*Tripordolide B* (**2**): colorless oil (MeOH); [α]${}_{D}^{25}$—35.1 (*c* 0.1, MeOH); IR (microscope) *ν*_max_ 3394, 2921, 1757, 1646, 1419, 1323, 1050, and 1027 cm^−1^; ^1^H NMR (DMSO-*d*_6_, 600 MHz) and ^13^C NMR (DMSO-*d*_6_, 150 MHz), see Table 1; HRESIMS *m*/*z* 399.1052 [M + Na]^+^ (calculated for C_19_H_20_NaO_7_, 399.1050). (Supplementary Materials).

*Tripordolide C* (**3**): colorless oil (MeOH); [α]${}_{D}^{25}$—77.3 (*c* 0.1, MeOH); UV (MeOH) *λ*_max_ (log *ε*) 220 (4.02) nm; ^1^H NMR (DMSO-*d*_6_, 600 MHz) and ^13^C NMR (DMSO-*d*_6_, 150 MHz), see Table 1; HRESIMS *m*/*z* 401.1574 [M + Na]^+^ (calculated for C_20_H_26_NaO_7_, 401.1571). (Supplementary Materials).

### 3.5. Biological Activities

The inhibitory effects of three compounds on NO production in LPS-induced RAW264.7 macrophage were evaluated. Dexamethasone was used a positive control. Concrete operation method was according to described in the literature [17].

## 4. Conclusions

In this research, no more than 30 18(4→3)-*abeo*-abietanes were isolated from the plant. Phytochemical studies on *T. wilfordii* have led to three *abeo*-abietanoids, a new natural product, and two new compounds, named tripordolides A–C (**1**–**3**). Tripordolide A (**1**) was reported in a patent as a synthetic product, but none of the spectra data was used as reference [18]. Its structure was elucidated on the basis of their spectroscopic analysis, and its absolute configuration was confirmed by an X-ray crystallographic analysis using anomalous scattering of Cu *K*α radiation. Notably, tripordolide A (**1**) was the first example of 18(4→3)-*abeo*-abietanes, which possessed 7/13 oxygen bridges in the natural products. Compounds **1** and **3** showed moderate inhibitory activities against NO production in LPS-induced RAW 264.7 macrophage in vitro.

## Figures and Tables

**Table 1:** ^1^H and ^13^C NMR data of compounds **1**–**3**.

NO.	1		2		3	
	*δ*_H_^a^ (mult., *J*/Hz)	*δ*_C_^b^	*δ*_H_^c^ (mult., *J*/Hz)	*δ*_C_^d^	*δ*_H_^c^ (mult., *J*/Hz)	*δ*_C_^d^
**1**	1.59, m; 1.89, m	27.5	7.22, d (8.0)	124.6	1.34, m; 2.18, m	29.8
**2**	2.03, m; 2.15, m	17.1	7.70, d (8.0)	123.1	2.12, m; 2.25, m	17.4
**3**		122.8		124.4		122.8
**4**		164.8		146.5		164.0
**5**	2.49, m	35.1		128.7	2.54, m	40.8
**6**	1.64, m; 1.53, m	24.6	2.50, m; 2.50, m	25.0	1.68, m; 2.06, m	30.2
**7**	3.96, d (2.8)	74.3	3.62, t (1.9)	58.3	4.22, m	70.6
**8**		75.9		60.4		131.7
**9**		76.0		60.7		137.8
**10**		40.2		138.5		35.6
**11**	6.12, d (overlap)	134.0	3.68, d (5.0)	66.7	4.45, br d (8.0)	62.0
**12**	6.10, d (overlap)	131.1	4.10, m	69.7	3.34, d (overlap)	55.9
**13**		82.7		74.9		61.9
**14**	3.99, d (6.0)	77.6	3.58, dd (7.8, 5.2)	74.8	4.47, d (4.4)	67.5
**15**		70.5		73.6		69.6
**16**	1.12, s	24.3	1.23, s	25.2	1.27, s	27.4
**17**	1.12, s	27.4	1.41, s	26.8	1.26, s	26.1
**18**		173.5		170.4		173.5
**19**	4.77, m	70.4	5.42, m	68.9	4.82, m	70.3
**20**	1.00, s	13.1			1.05, s	21.5
**7-OH**					5.34, d (5.4)	
**8-OH**	4.86, s					
**9-OH**	4.03, s					
**11-OH**					5.02, d (8.0)	
**14-OH**	4.90, d(6.0 )				4.86, d (4.4)	
**15-OH**	4.15, s				4.17, s	

^a^ In DMSO-*d*6 (500 MHz); ^b^ In DMSO-*d*6 (125 MHz). ^c^ In DMSO-*d*6 (600 MHz); ^d^ In DMSO-*d*6 (150 MHz).

## Supplementary Materials

The following are available online, copies of ^1^H, ^13^C NMR, DEPT, ^1^H, ^1^H COSY, HSQC, HMBC, NOESY and HRESIMS spectra of new compounds. This material is available free of charge online.
