# Supplementary material for: New 18(4→3)-Abeo-Abietanoids from Tripterygium wilfordii
Source: Molecules. 2018 Sep 26;23(10):2467. doi: 10.3390/molecules23102467 (PMC6222710; doi:10.3390/molecules23102467)
Supplement: Supplementary file 1 [file molecules-23-02467-s001.pdf]

New *Abeo*-abietanoids from *Tripterygium wilfordii*

Lin Ni[1,2], Yingda Zang, Jingzhi Yang, Chuang-jun Li[1], Jie Ma[1], and Dong-ming Zhang[1*]

Supplementary Materials

[1]State Key Laboratory of Bioactive Substance and Function of Natural Medicines, Institute of Materia Medica, Chinese Academy of Medical Sciences and Peking Union Medical College, Beijing 100050, People’s Republic of China.

[2]College of Plant Protection, Fujian Agriculture and Forestry University, Fuzhou 350002, Fujian Province, People’s Republic of China.

*Corresponding author. E-mail: zhangdm@imm.ac.cn; Tel/Fax: +86-10-63165227.

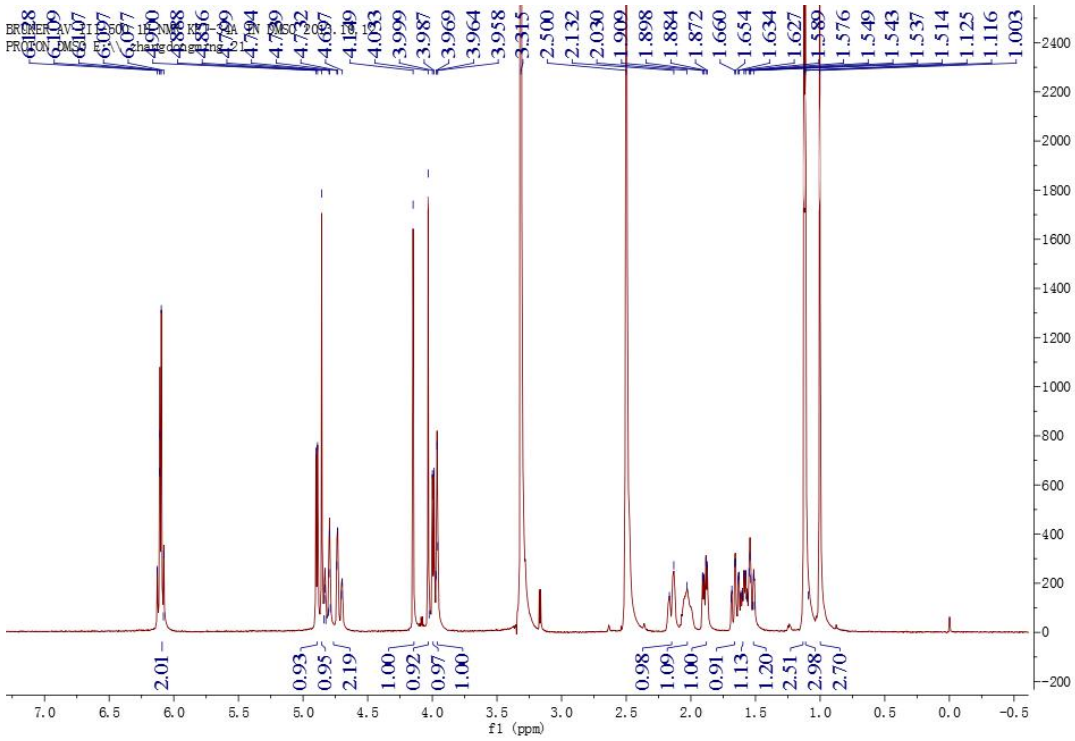

^{1}H NMR spectrum of compound **1**

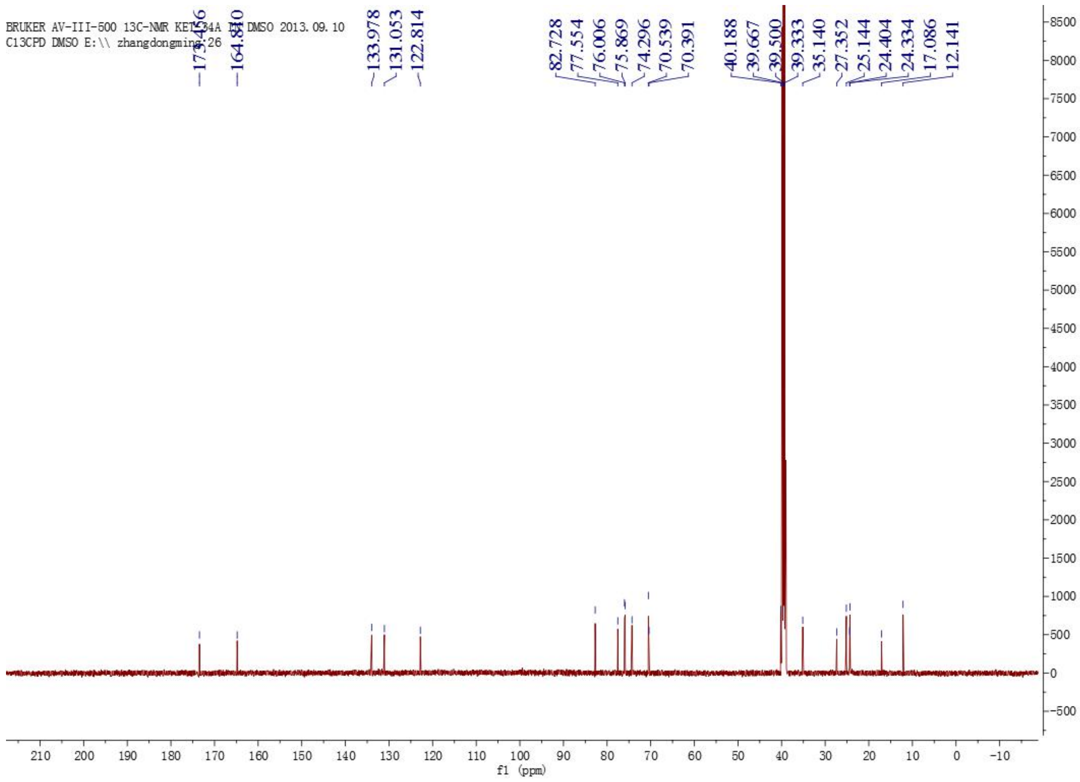

^{13}C NMR spectrum of compound **1**

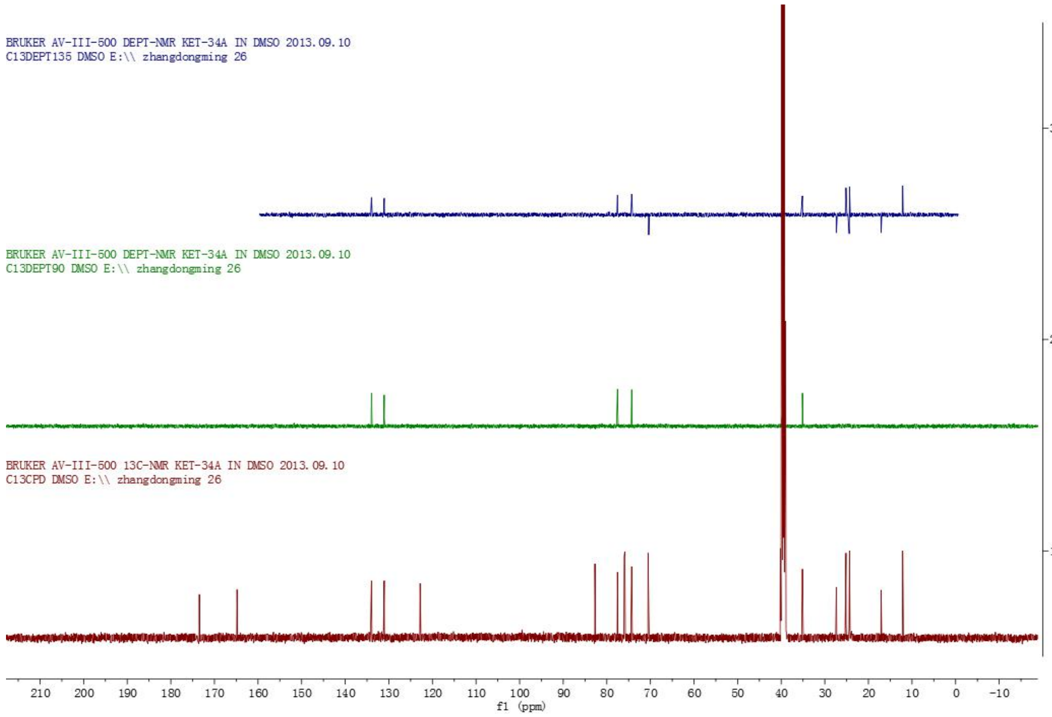

DEPT spectrum of compound **1**

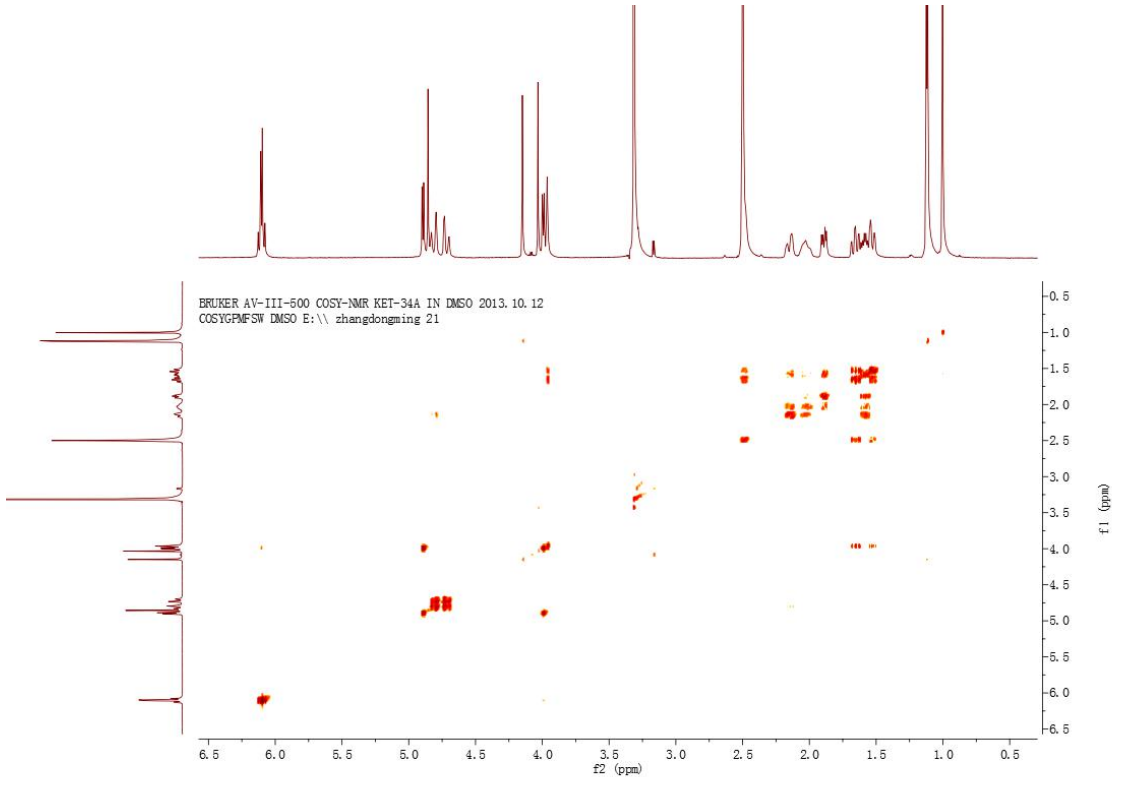

$^{1}H,^{1}H$ COSY spectrum of compound **1**

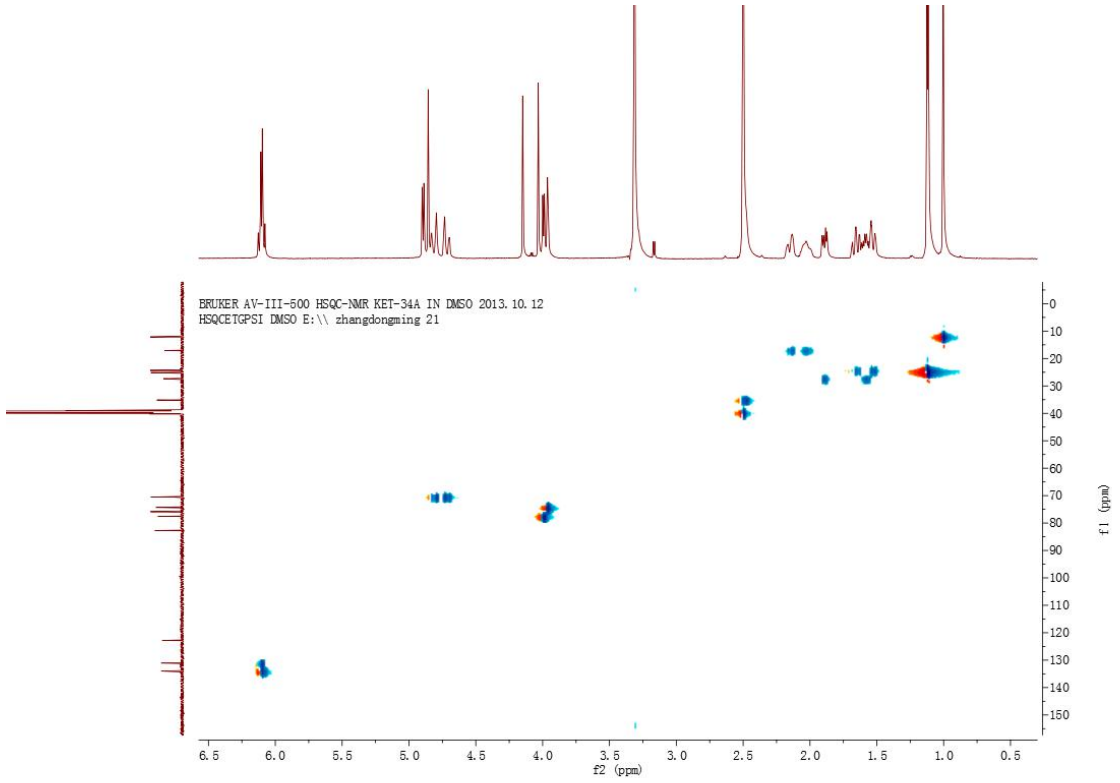

HSQC spectrum of compound **1**

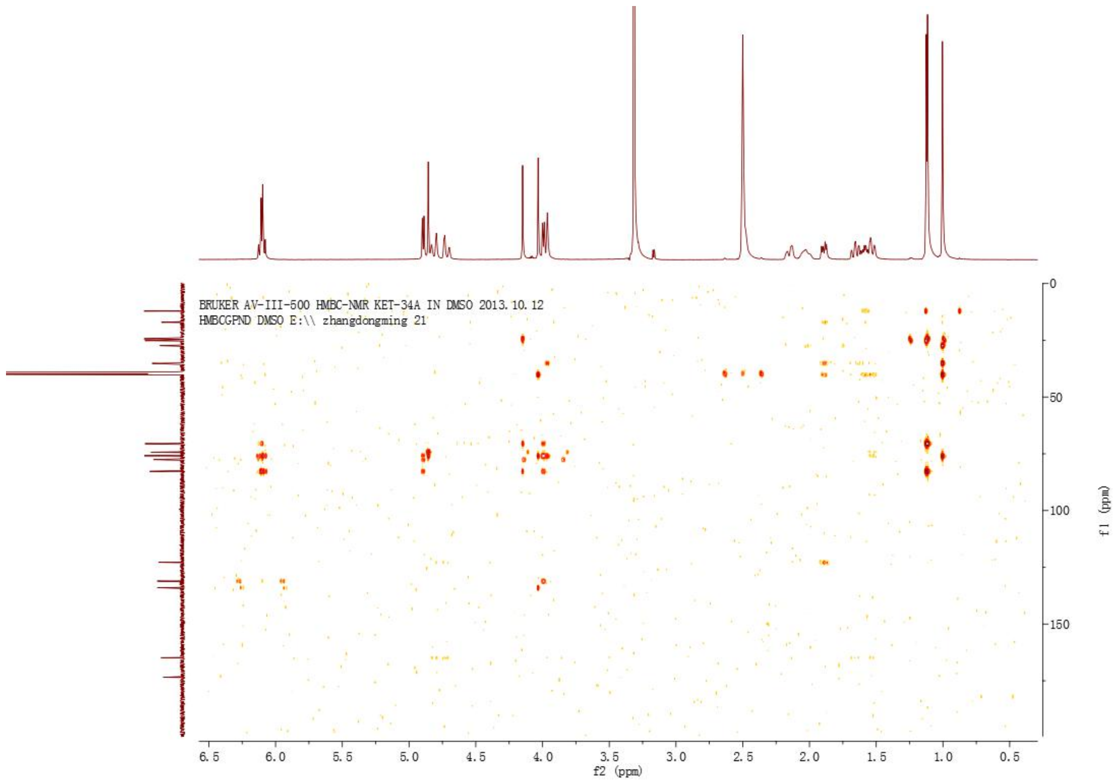

HMBC spectrum of compound **1**

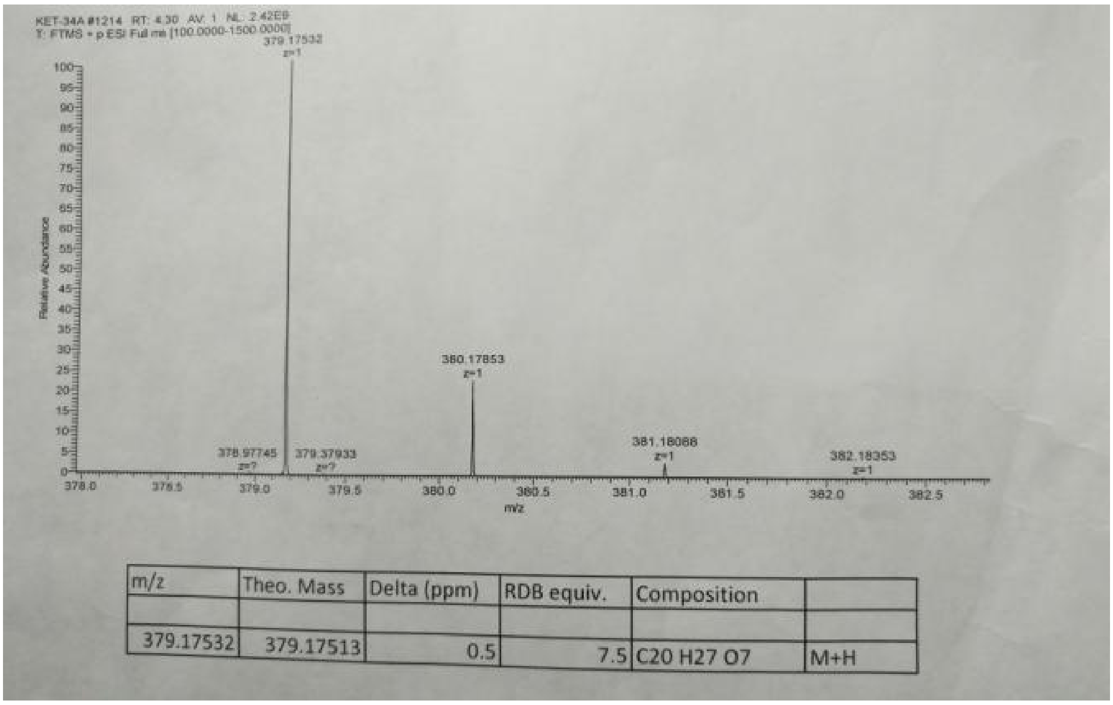

m/z	Theo. Mass	Delta (ppm)	RDB equiv.	Composition	
379.17532	379.17513	0.5	7.5	C20 H27 O7	M+H

HRESIMS spectrum of compound **1**

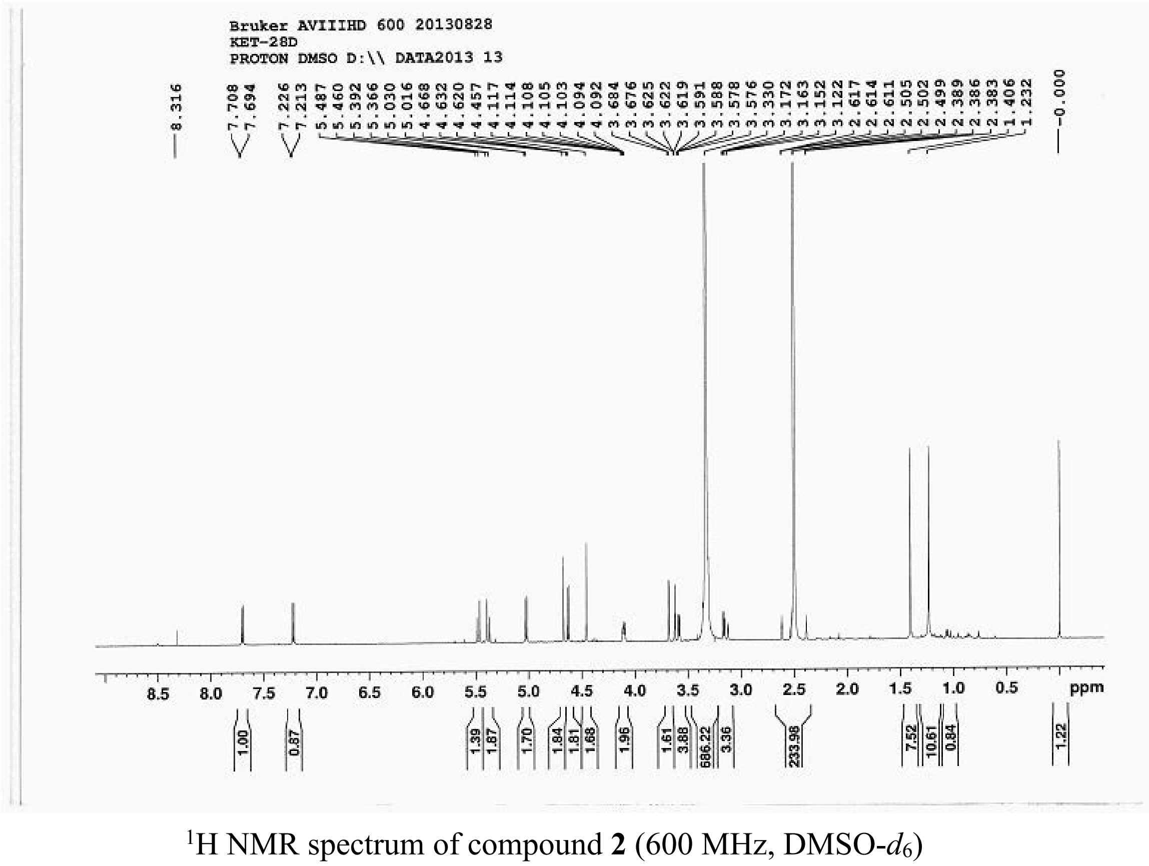

^{1}H NMR spectrum of compound **2** (600 MHz, DMSO-d_6)

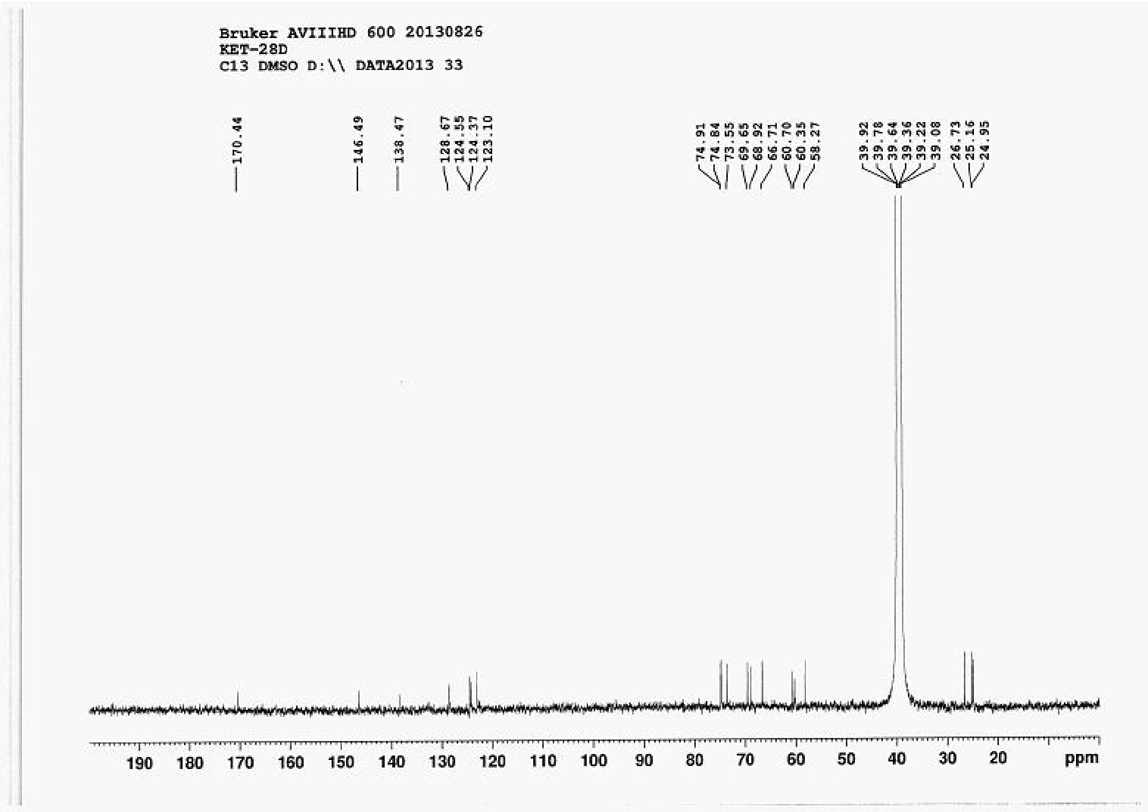

^{13}C NMR spectrum of compound **2** (150 MHz, DMSO-d_6)

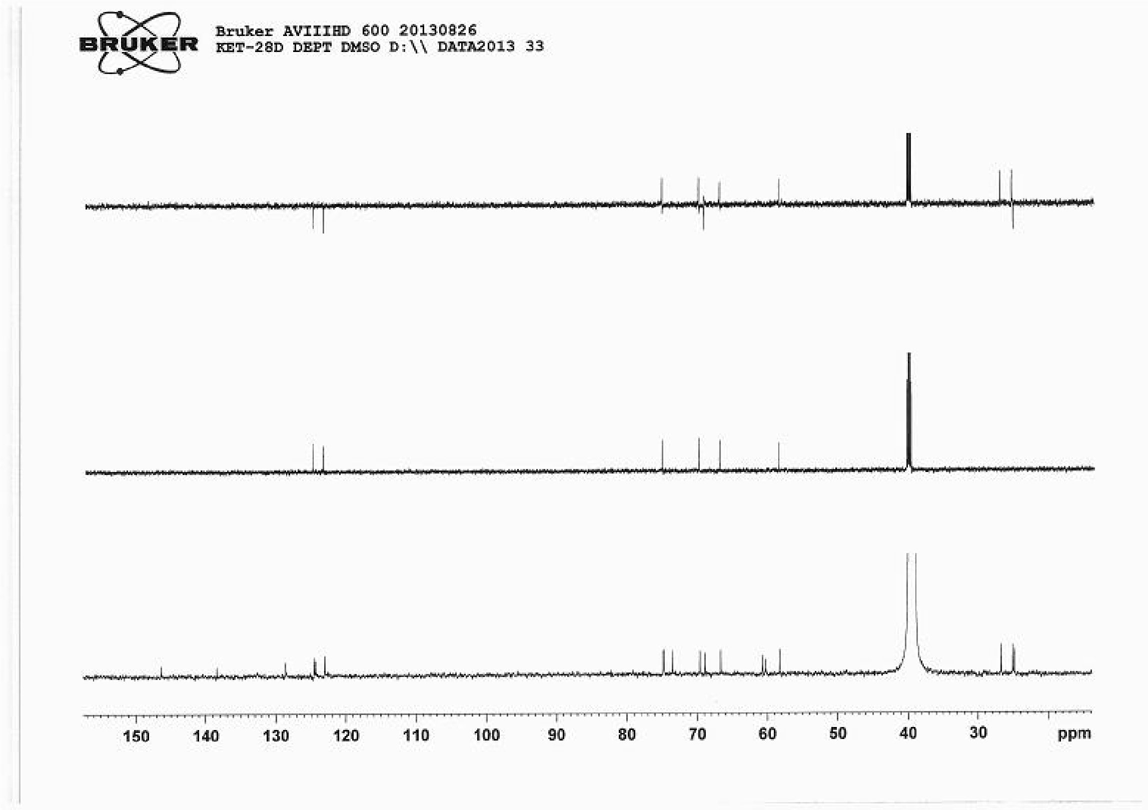

DEPT spectrum of compound **2** (150 MHz, DMSO-d_6)

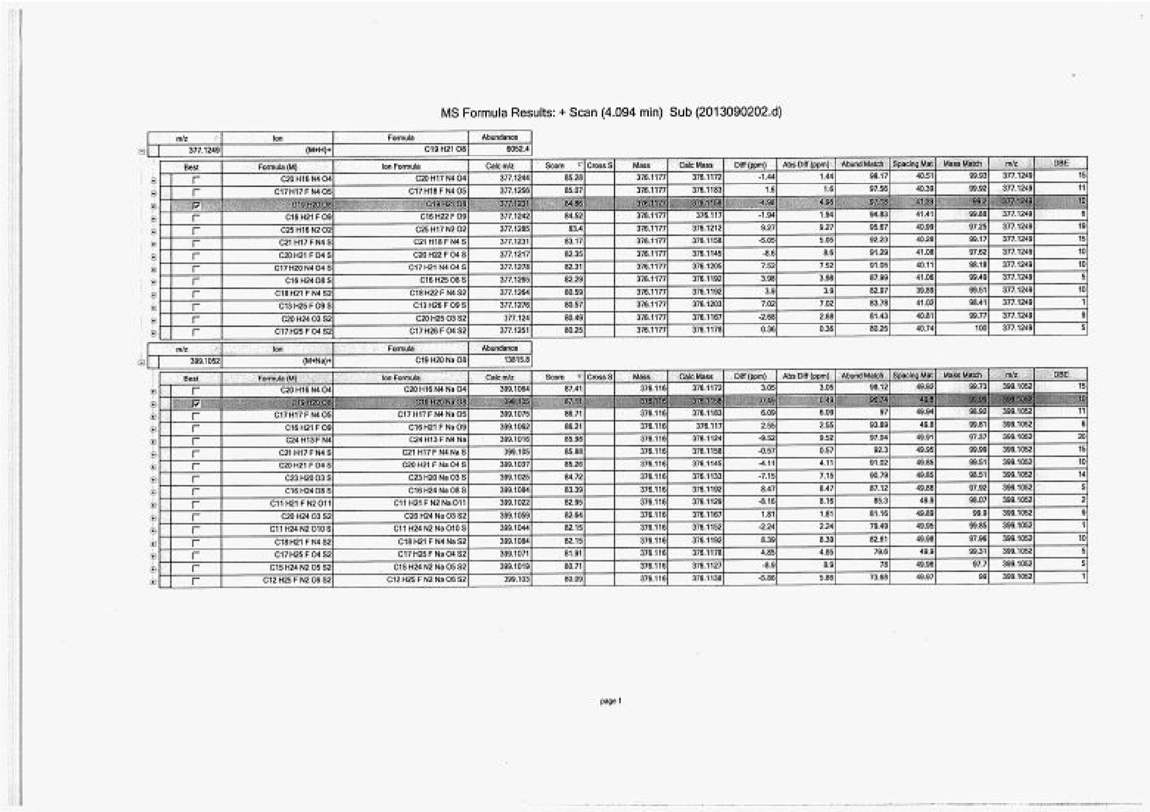

MS Formula Results: + Scan (4.094 min) Sub (2013090202.d)

m/z	Ion	Formula	Abundance
377.1249	(M+H)+	C19 H21 O8	8052.4

Best	Formula (M)	Ion Formula	Calc m/z	Score	Cross S	Mass	Calc Mass	Diff (ppm)	Abs Diff (ppm)	Abund Match	Spacing Mat	Mass Match	m/z	DBE
	C20 H16 N4 O4	C20 H17 N4 O4	377.1244	85.28		376.1177	376.1172	-1.44	1.44	98.17	40.51	99.93	377.1249	15
	C17 H17 F N4 O5	C17 H18 F N4 O5	377.1256	85.07		376.1177	376.1183	1.6	1.6	97.56	40.38	99.92	377.1249	11
✓	C19 H20 O8	C19 H21 O8	377.1231	84.86		376.1177	376.1158	-4.98	4.98	[illegible]	[illegible]	99.2	377.1249	10
	C16 H21 F O9	C16 H22 F O9	377.1242	84.52		376.1177	376.117	-1.94	1.94	94.83	41.41	99.88	377.1249	6
	C25 H16 N2 O2	C25 H17 N2 O2	377.1285	83.4		376.1177	376.1212	9.27	9.27	95.87	40.98	97.25	377.1249	19
	C21 H17 F N4 S	C21 H18 F N4 S	377.1231	83.17		376.1177	376.1158	-5.05	5.05	92.23	40.28	99.17	377.1249	15
	C20 H21 F O4 S	C20 H22 F O4 S	377.1217	82.35		376.1177	376.1145	-8.6	8.6	91.29	41.08	97.62	377.1249	10
	C17 H20 N4 O4 S	C17 H21 N4 O4 S	377.1278	82.31		376.1177	376.1205	7.52	7.52	91.05	40.11	98.18	377.1249	10
	C16 H24 O8 S	C16 H25 O8 S	377.1265	82.29		376.1177	376.1192	3.98	3.98	87.89	41.06	99.48	377.1249	5
	C18 H21 F N4 S2	C18 H22 F N4 S2	377.1264	80.59		376.1177	376.1192	3.9	3.9	82.87	39.88	99.51	377.1249	10
	C13 H25 F O9 S	C13 H26 F O9 S	377.1276	80.57		376.1177	376.1203	7.02	7.02	83.78	41.02	98.41	377.1249	1
	C20 H24 O3 S2	C20 H25 O3 S2	377.124	80.49		376.1177	376.1167	-2.68	2.68	81.43	40.81	99.77	377.1249	9
	C17 H25 F O4 S2	C17 H26 F O4 S2	377.1251	80.25		376.1177	376.1178	0.36	0.36	80.25	40.74	100	377.1249	5

m/z	Ion	Formula	Abundance
399.1052	(M+Na)+	C19 H20 Na O8	13815.8

Best	Formula (M)	Ion Formula	Calc m/z	Score	Cross S	Mass	Calc Mass	Diff (ppm)	Abs Diff (ppm)	Abund Match	Spacing Mat	Mass Match	m/z	DBE
	C20 H16 N4 O4	C20 H16 N4 Na O4	399.1064	87.41		376.116	376.1172	3.05	3.05	98.12	49.99	99.73	399.1052	15
✓	C19 H20 O8	C19 H20 Na O8	399.105	[illegible]		376.116	376.1158	[illegible]	0.49	96.74	49.9	[illegible]	399.1052	10
	C17 H17 F N4 O5	C17 H17 F N4 Na O5	399.1075	86.71		376.116	376.1183	6.09	6.09	97	49.94	98.90	399.1052	11
	C16 H21 F O9	C16 H21 F Na O9	399.1062	86.21		376.116	376.117	2.56	2.55	93.89	49.8	99.81	399.1052	6
	C24 H13 F N4	C24 H13 F N4 Na	399.1016	85.98		376.116	376.1124	-9.52	9.52	97.94	49.91	97.37	399.1052	20
	C21 H17 F N4 S	C21 H17 F N4 Na S	399.105	85.88		376.116	376.1158	-0.57	0.57	92.3	49.95	99.98	399.1052	15
	C20 H21 F O4 S	C20 H21 F Na O4 S	399.1037	85.26		376.116	376.1145	-4.11	4.11	91.02	49.85	99.51	399.1052	10
	C23 H20 O3 S	C23 H20 Na O3 S	399.1025	84.72		376.116	376.1133	-7.15	7.15	90.79	49.85	98.51	399.1052	14
	C16 H24 O8 S	C16 H24 Na O8 S	399.1084	83.39		376.116	376.1192	8.47	8.47	87.12	49.88	97.92	399.1052	5
	C11 H21 F N2 O11	C11 H21 F N2 Na O11	399.1022	82.95		376.116	376.1129	-8.16	8.16	85.3	49.8	98.07	399.1052	2
	C20 H24 O3 S2	C20 H24 Na O3 S2	399.1059	82.64		376.116	376.1167	1.81	1.81	81.16	49.89	99.8	399.1052	9
	C11 H24 N2 O10 S	C11 H24 N2 Na O10 S	399.1044	82.15		376.116	376.1152	-2.24	2.24	78.49	49.95	99.85	399.1052	1
	C18 H21 F N4 S2	C18 H21 F N4 Na S2	399.1084	82.15		376.116	376.1192	8.39	8.39	82.81	49.98	97.96	399.1052	10
	C17 H25 F O4 S2	C17 H25 F Na O4 S2	399.1071	81.91		376.116	376.1178	4.85	4.85	79.6	49.9	99.31	399.1052	5
	C15 H24 N2 O6 S2	C15 H24 N2 Na O6 S2	399.1019	80.71		376.116	376.1127	-8.9	8.9	78	49.98	97.7	399.1052	5
	C12 H25 F N2 O6 S2	C12 H25 F N2 Na O6 S2	399.103	80.20		376.116	376.1138	-5.88	5.88	73.88	49.97	98	399.1052	1

page 1

HRESIMS spectrum of compound **2**

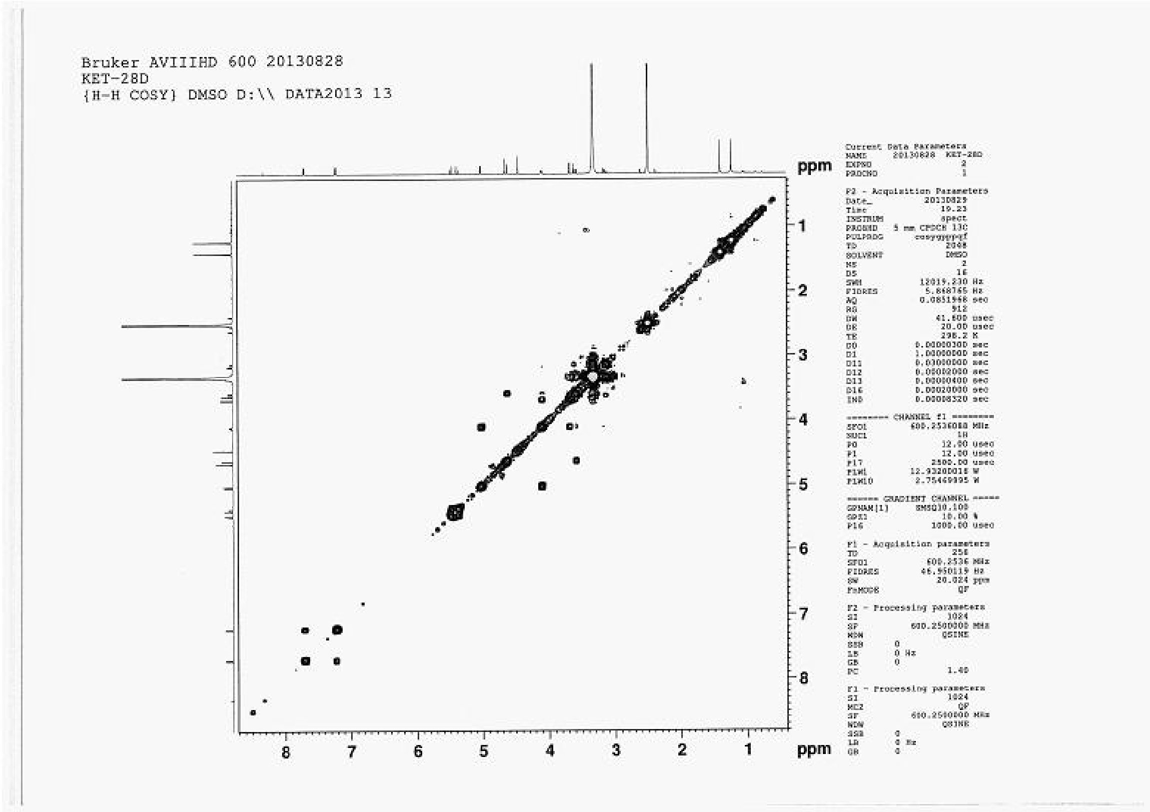

^{1}H,^{1}H COSY spectrum of compound **2**

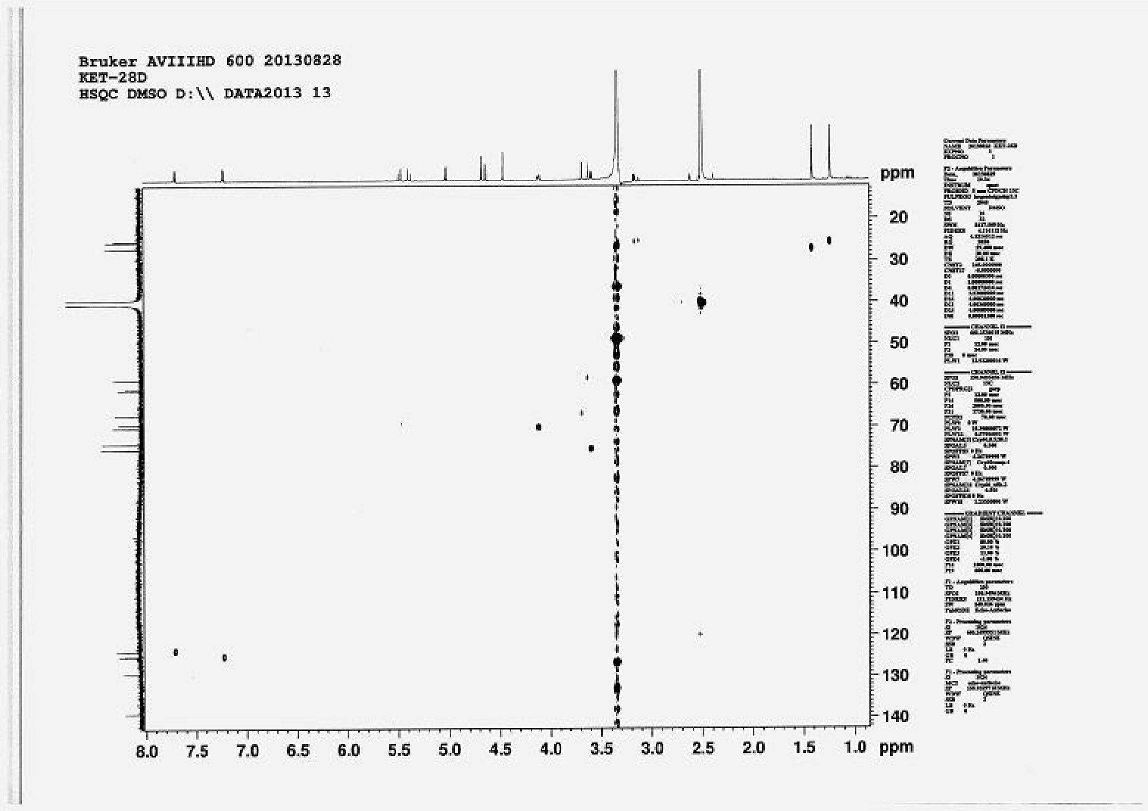

HSQC spectrum of compound **2**

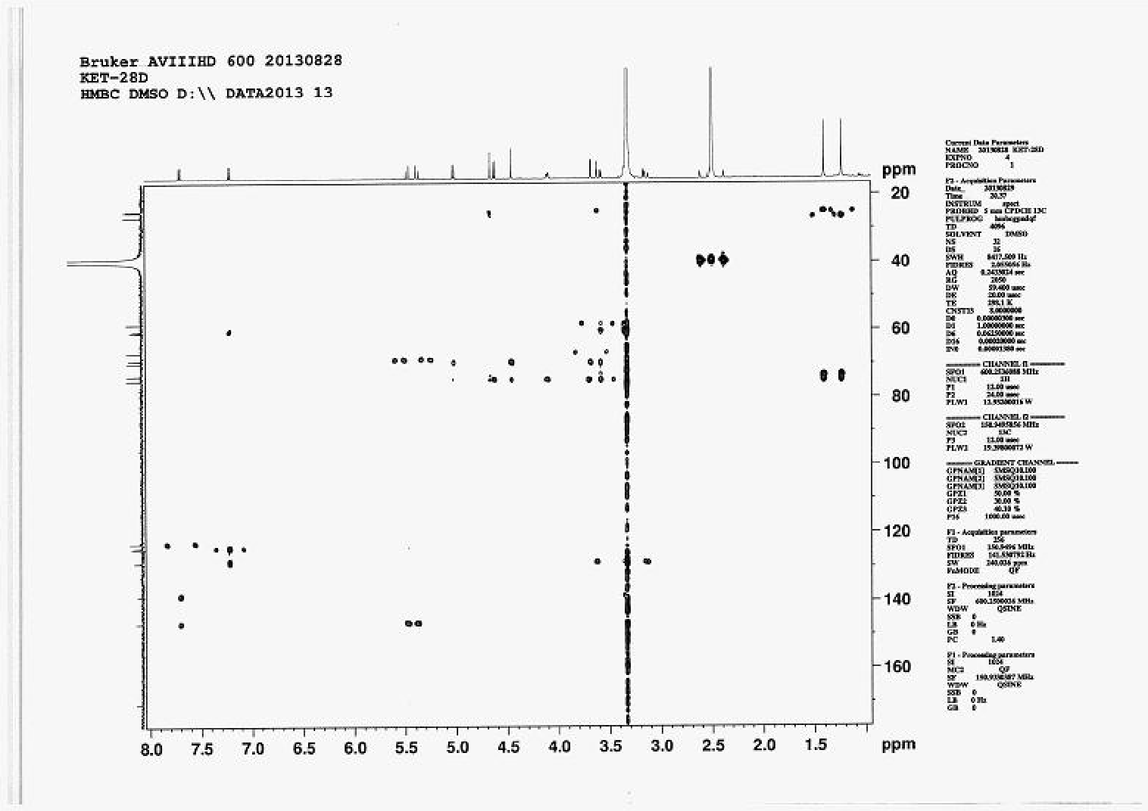

HMBC spectrum of compound **2**

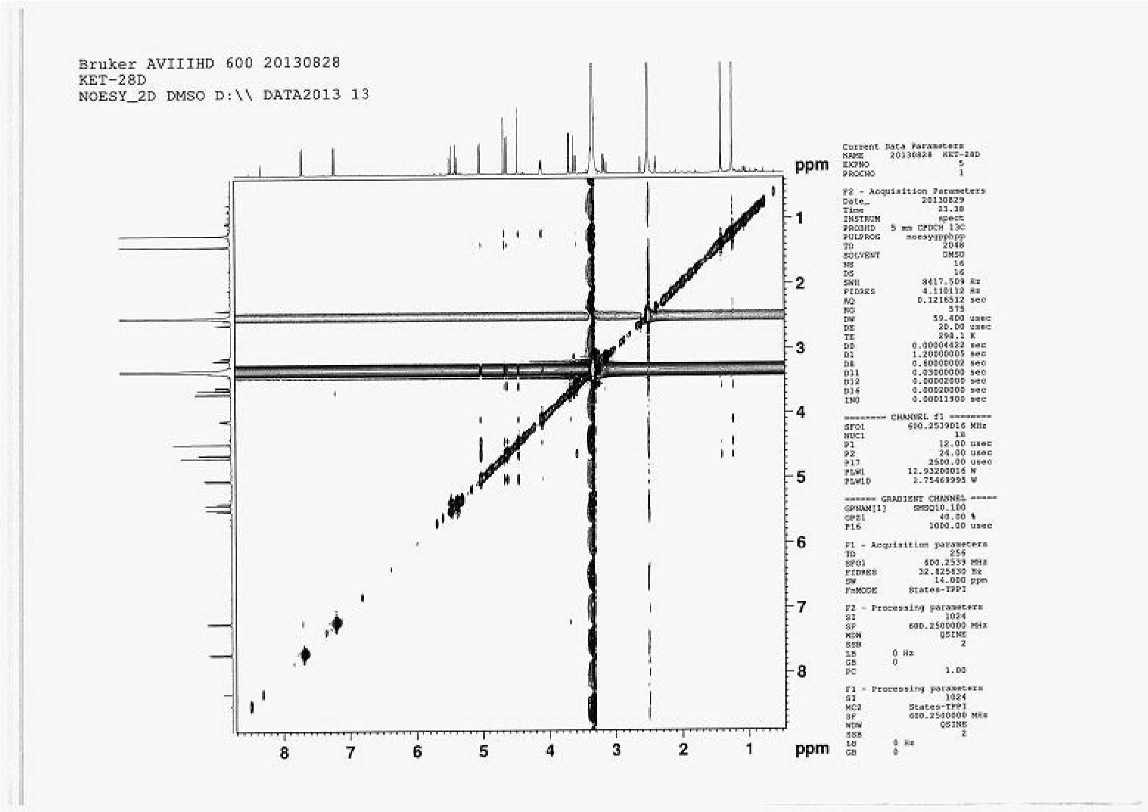

NOESY spectrum of compound **2**

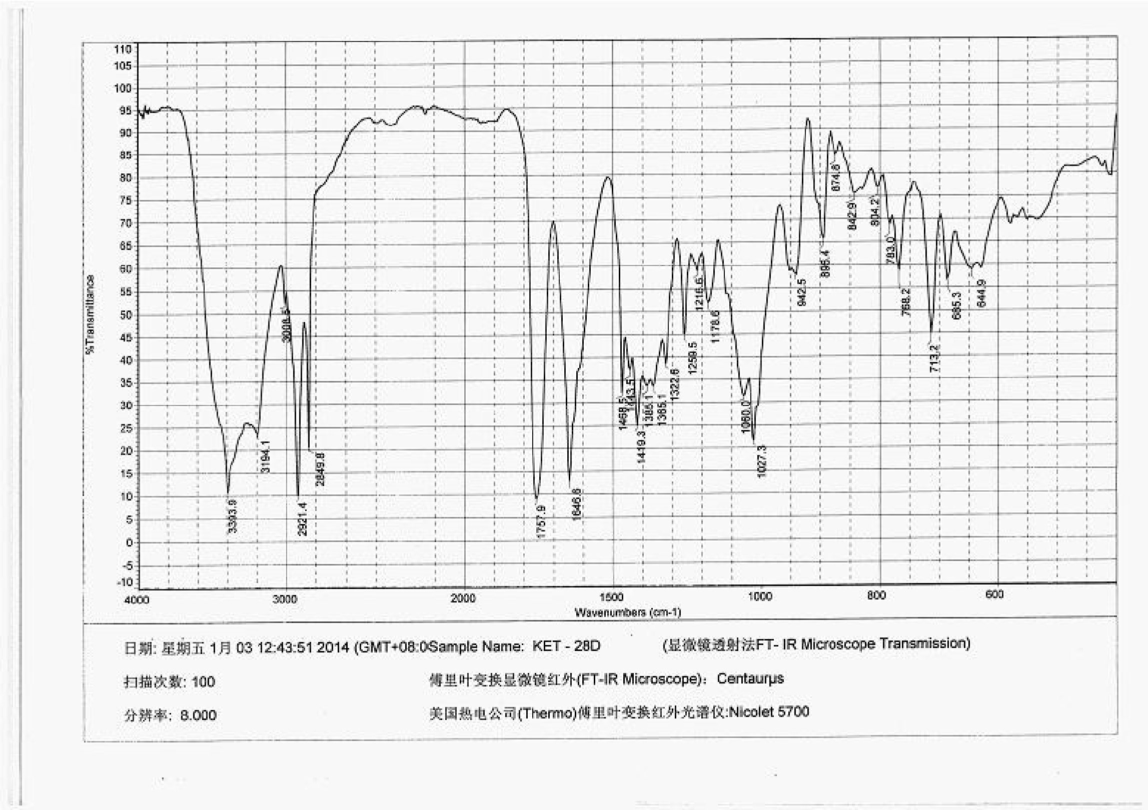

IR spectrum of compound **2**

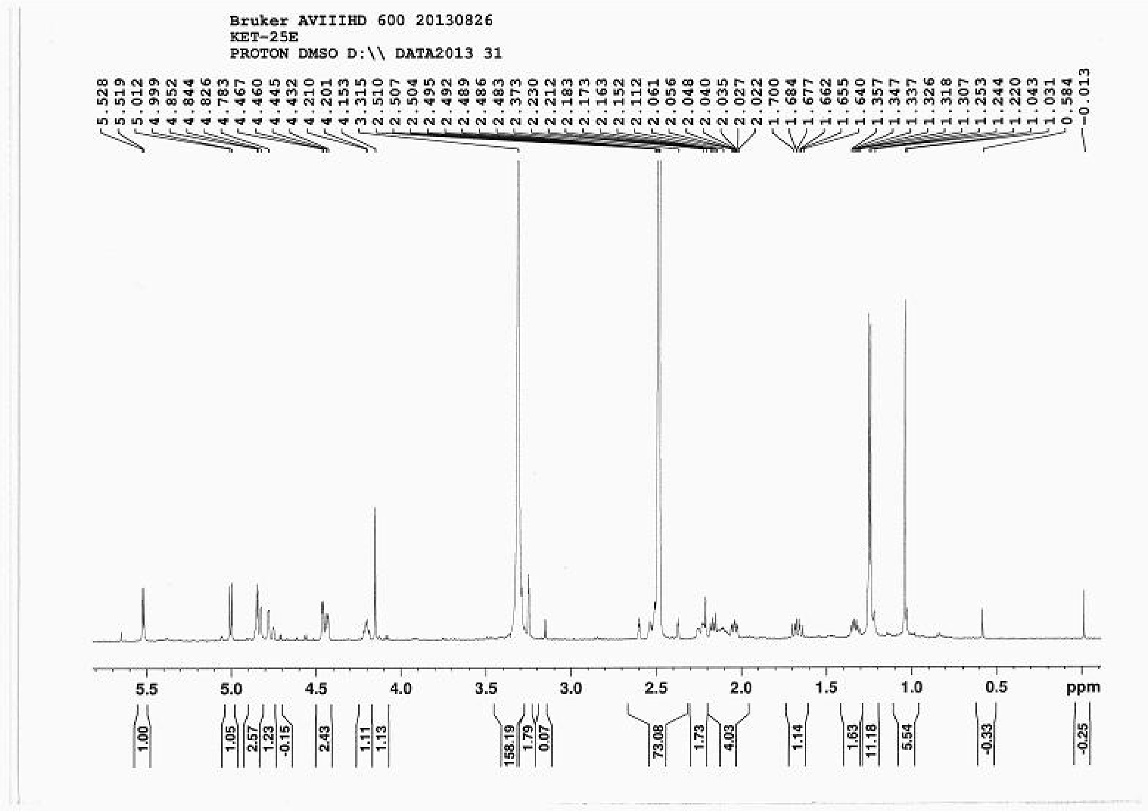

^{1}H NMR spectrum of compound **3** (600 MHz, DMSO-d_6)

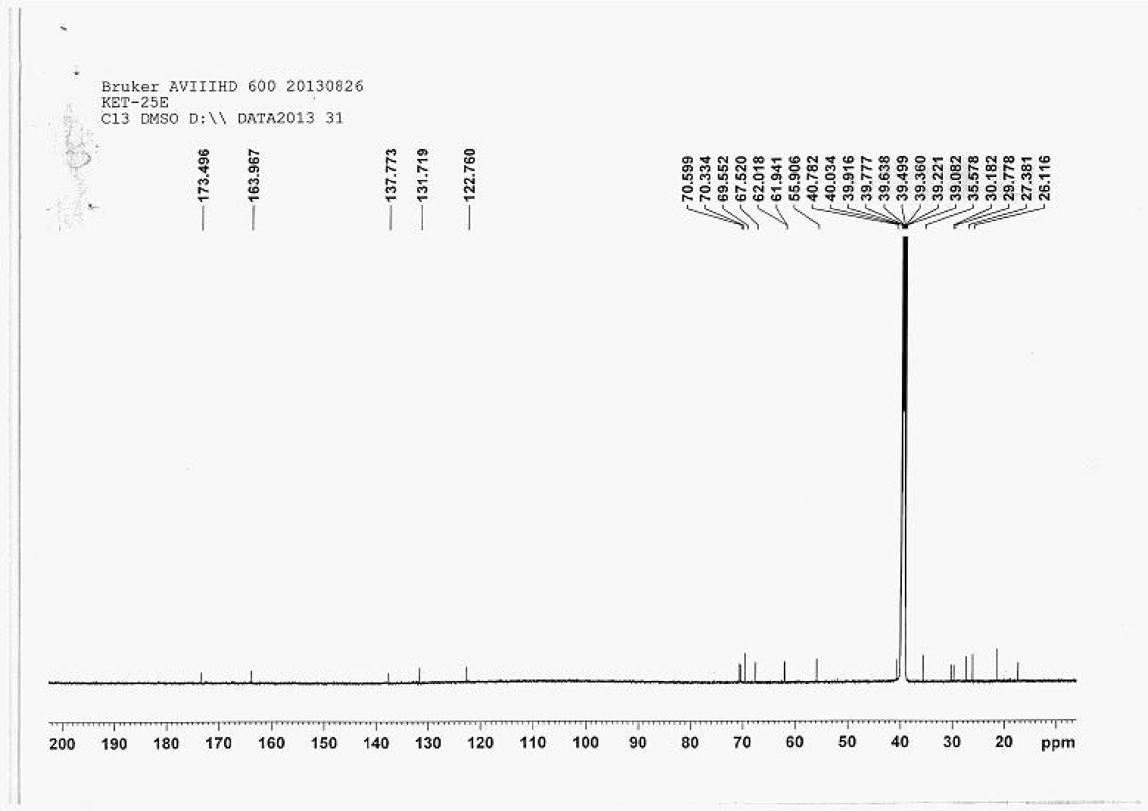

^{13}C NMR spectrum of compound **3** (150 MHz, DMSO-d_6)

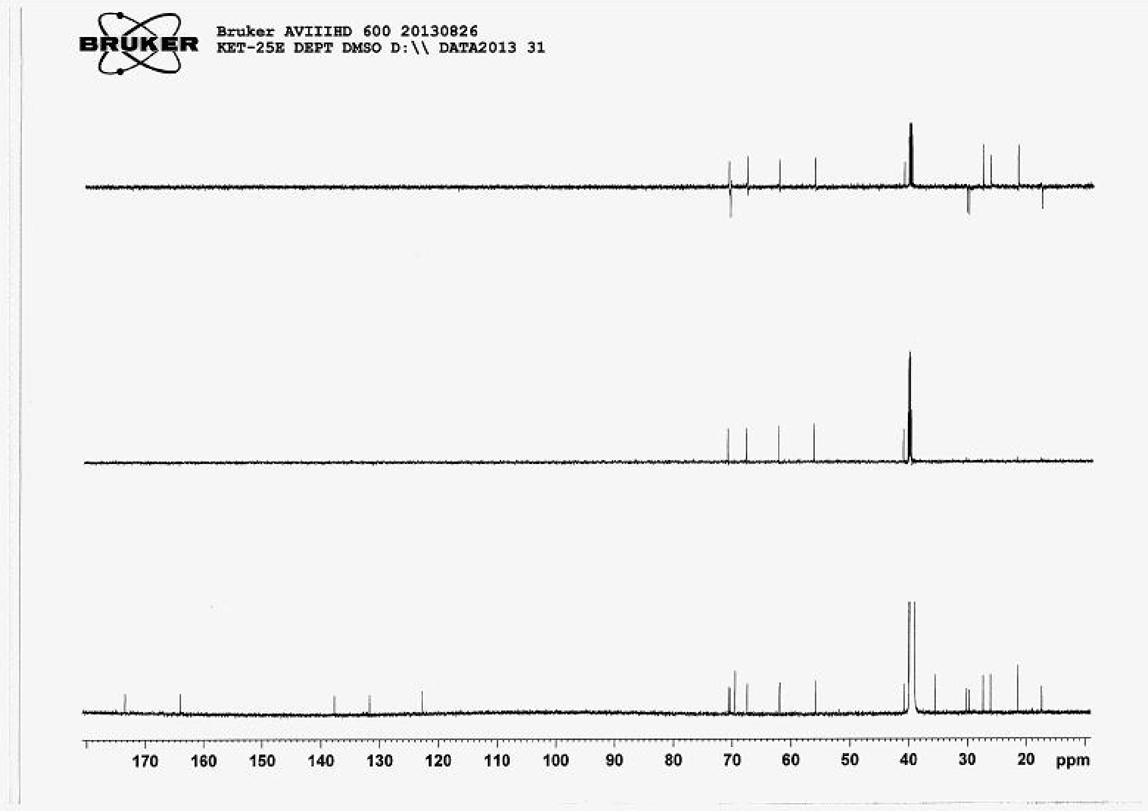

DEPT spectrum of compound **3** (150 MHz, DMSO-d_6)

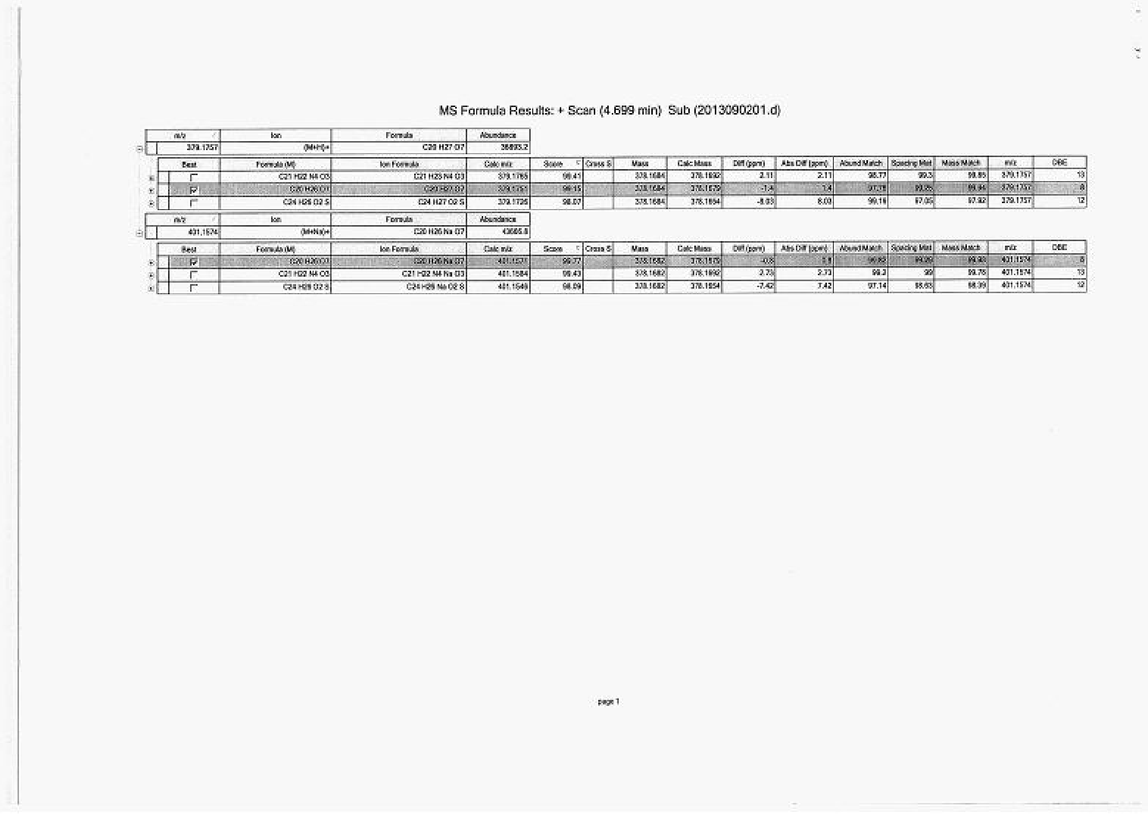

MS Formula Results: + Scan (4.699 min) Sub (2013090201.d)

m/z	Ion	Formula	Abundance
379.1757	(M+H)+	C20 H27 O7	36893.2

Best	Formula (M)	Ion Formula	Calc m/z	Score	Cross S	Mass	Calc Mass	Diff (ppm)	Abs Diff (ppm)	Abund Match	Spacing Mat	Mass Match	m/z	DBE
☐	C21 H22 N4 O3	C21 H23 N4 O3	379.1765	98.41		378.1684	378.1692	2.11	2.11	98.77	99.3	98.85	379.1757	13
☑	C20 H26 O7	C20 H27 O7	[illegible]	98.15		378.1684	378.1679	-1.4	1.4	[illegible]	[illegible]	[illegible]	379.1757	8
☐	C24 H26 O2 S	C24 H27 O2 S	379.1726	98.07		378.1684	378.1654	-8.03	8.03	99.16	97.05	97.92	379.1757	12

m/z	Ion	Formula	Abundance
401.1574	(M+Na)+	C20 H26 Na O7	43686.8

Best	Formula (M)	Ion Formula	Calc m/z	Score	Cross S	Mass	Calc Mass	Diff (ppm)	Abs Diff (ppm)	Abund Match	Spacing Mat	Mass Match	m/z	DBE
☑	C20 H26 O7	C20 H26 Na O7	[illegible]	98.77		378.1682	378.1679	-0.8	0.8	[illegible]	[illegible]	[illegible]	401.1574	8
☐	C21 H22 N4 O3	C21 H22 N4 Na O3	401.1584	98.43		378.1682	378.1692	2.73	2.73	98.2	99	98.78	401.1574	13
☐	C24 H26 O2 S	C24 H26 Na O2 S	401.1546	98.09		378.1682	378.1654	-7.42	7.42	97.14	98.65	98.39	401.1574	12

page 1

HRESIMS spectrum of compound **3**

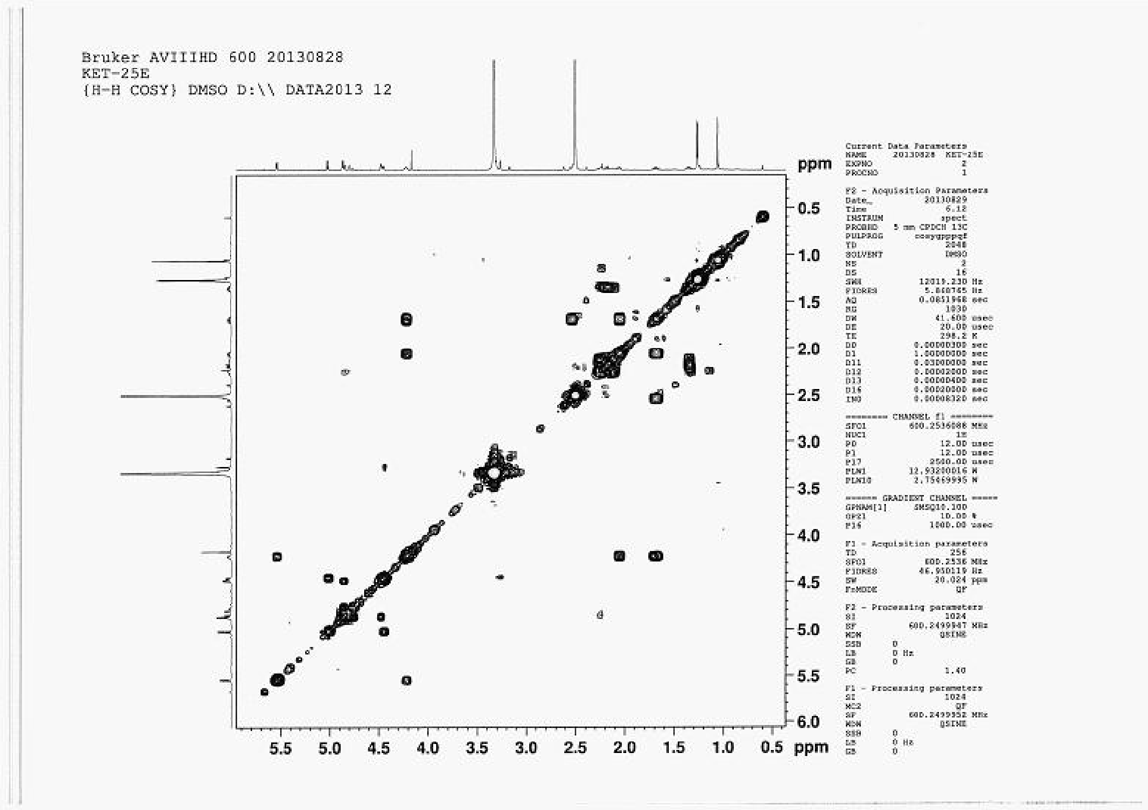

$^1H,^1H$ COSY spectrum of compound **3**

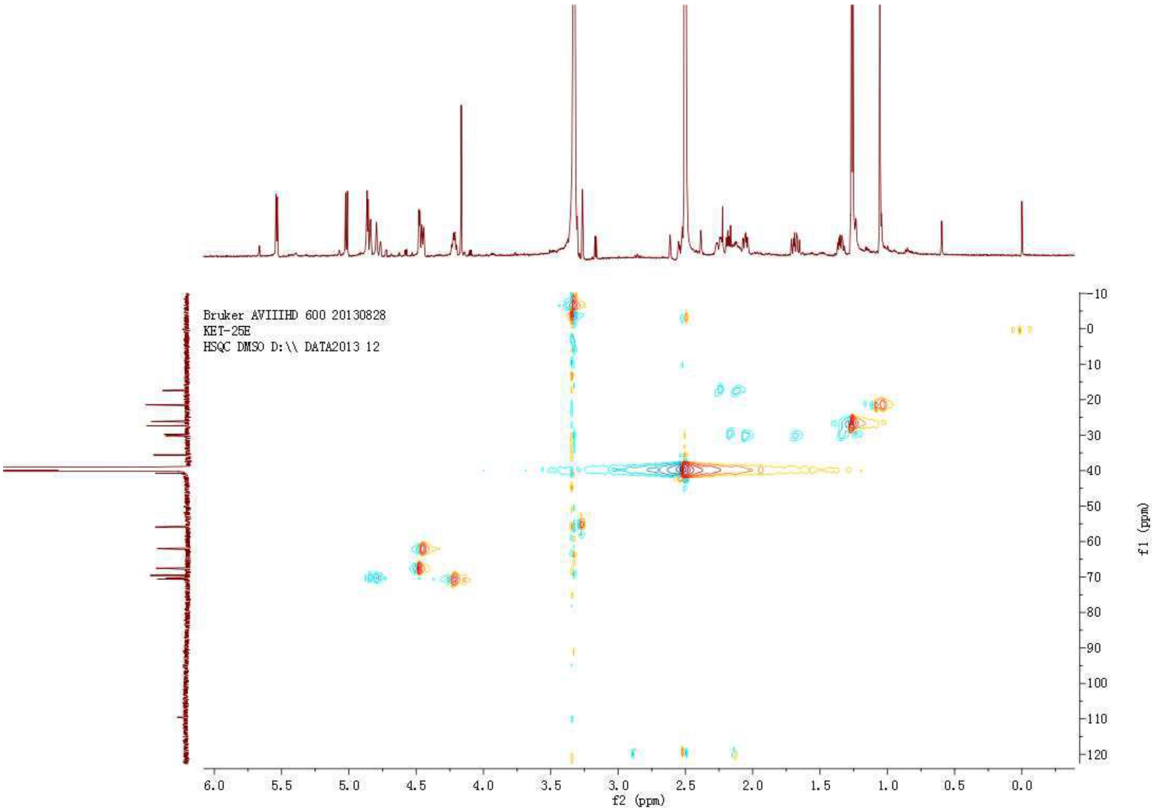

HSQC spectrum of compound **3**

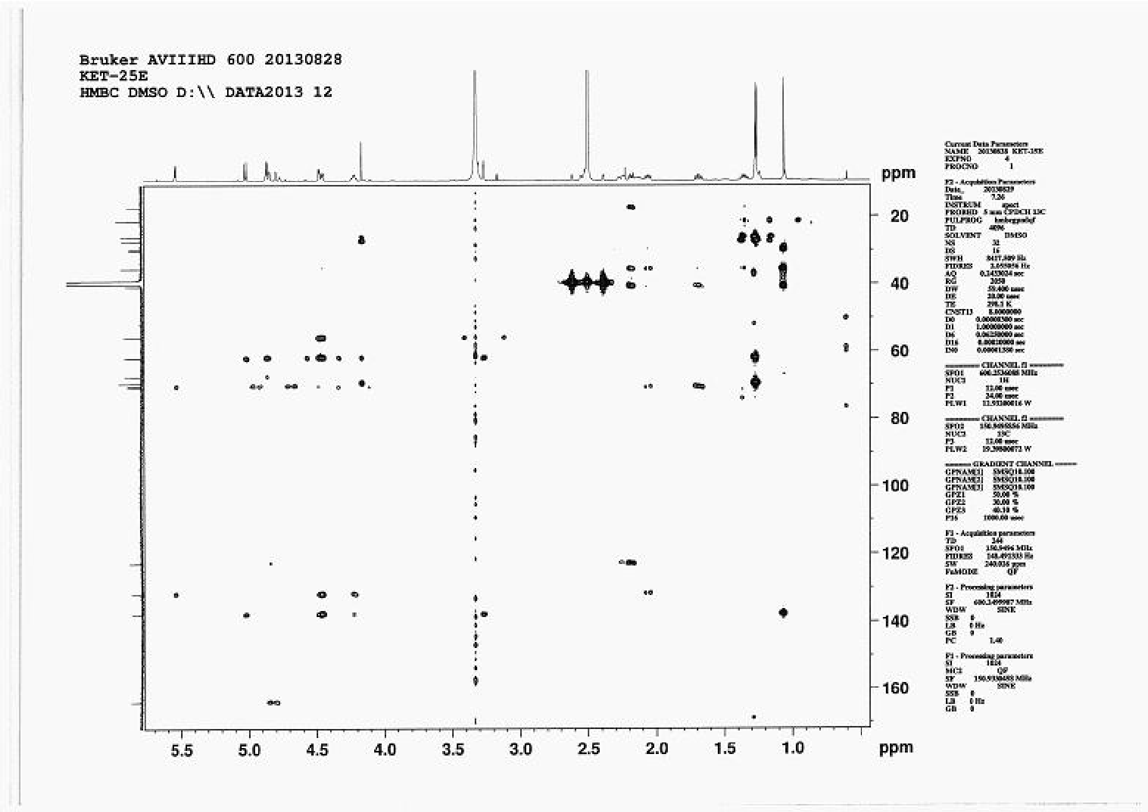

HMBC spectrum of compound **3**

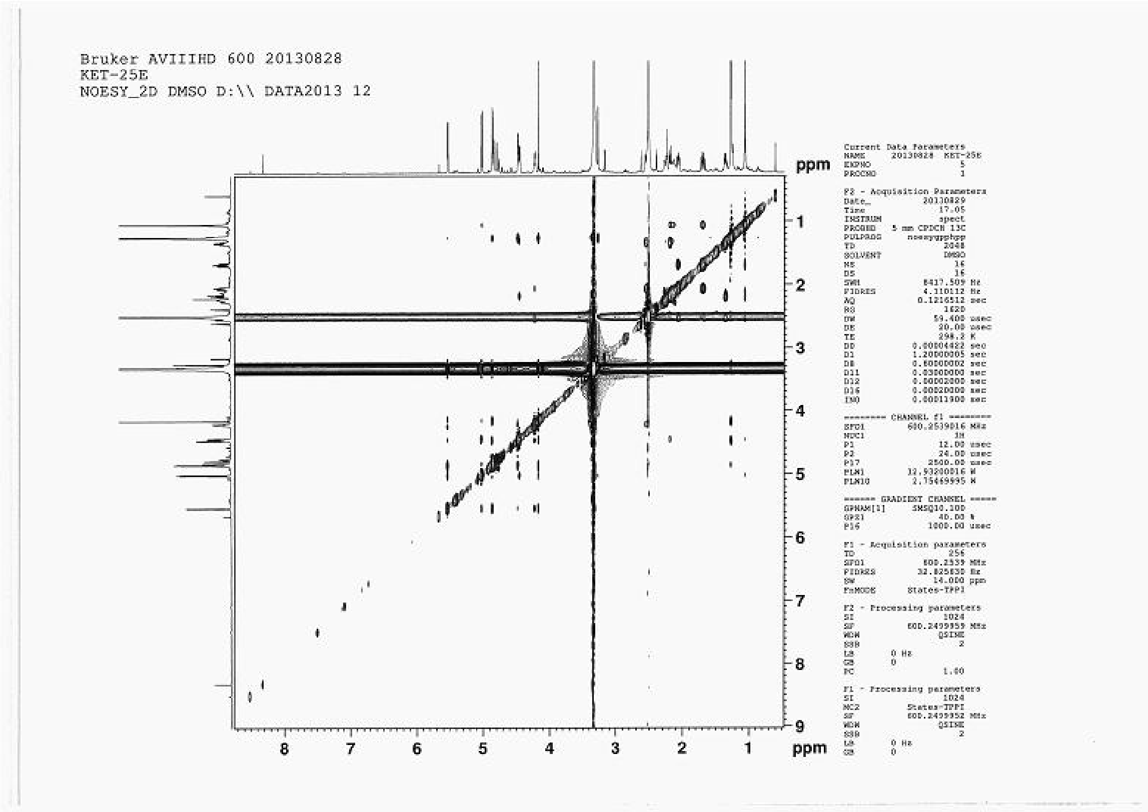

NOESY spectrum of compound **3**
